# Umbilical cord-derived mesenchymal stem cell sheets transplanted subcutaneously enhance cell retention and survival more than dissociated stem cell injections

**DOI:** 10.1186/s13287-023-03593-0

**Published:** 2023-12-10

**Authors:** Mitsuyoshi Nakao, Makoto Matsui, Kyungsook Kim, Nobuhiro Nishiyama, David W. Grainger, Teruo Okano, Hideko Kanazawa, Kenichi Nagase

**Affiliations:** 1 Faculty of Pharmacy, Keio University, 1-5-30 Shibakoen, Minato-ku, Tokyo, 105-8512 Japan; 2 Laboratory for Chemistry and Life Science, Institute of Innovative Research, Tokyo Institute of Technology, 4259 Nagatsuta-cho, Midori-ku, Yokohama, Kanagawa 226-8503 Japan; 3 Cell Sheet Tissue Engineering Center (CSTEC), Department of Molecular Pharmaceutics, University of Utah, Health Sciences, Salt Lake City, UT 84112 USA; 4 Department of Biomedical Engineering, University of Utah, Salt Lake City, UT 84112 USA

**Keywords:** Cell sheet transplantation, Cytokine expression, Engraftment, Temperature-responsive cell cultureware, Cell retention

## Abstract

**Background:**

Human umbilical cord-derived mesenchymal stem cell (hUC-MSC) sheets have recently attracted attention as an alternative approach to injected cell suspensions for stem cell therapy. However, cell engraftment and cytokine expression levels between hUC-MSC sheets and their cell suspensions in vivo have not yet been compared. This study compares hUC-MSC in vivo engraftment efficacy and cytokine expression for both hUC-MSC sheets and cell suspensions.

**Methods:**

hUC-MSC sheets were prepared using temperature-responsive cell culture; two types of hUC-MSC suspensions were prepared, either by enzymatic treatment (trypsin) or by enzyme-free temperature reduction using temperature-responsive cell cultureware. hUC-MSC sheets and suspensions were transplanted subcutaneously into ICR mice through subcutaneous surgical placement and intravenous injection, respectively. hUC-MSC sheet engraftment after subcutaneous surgical transplantation was investigated by in vivo imaging while intravenously injected cell suspensions were analyzing using in vitro organ imaging. Cytokine levels in both transplant site tissues and blood were quantified by enzyme-linked immunosorbent assay.

**Results:**

After subcutaneous transplant, hUC-MSC sheets exhibited longer engraftment duration than hUC-MSC suspensions. This was attributed to extracellular matrix (ECM) and cell–cell junctions retained in sheets but enzymatically altered in suspensions. hUC-MSC suspensions harvested using enzyme-free temperature reduction exhibited relatively long engraftment duration after intravenous injection compared to suspensions prepared using trypsin, as enzyme-free harvest preserved cellular ECM. High HGF and TGF-β1 levels were observed in sheet-transplanted sites compared to hUC-MSC suspension sites. However, no differences in human cytokine levels in murine blood were detected, indicating that hUC-MSC sheets might exert local paracrine rather than endocrine effects.

**Conclusions:**

hUC-MSC sheet transplantation could be a more effective cell therapeutic approach due to enhanced engraftment and secretion of therapeutic cytokines over injected hUC-MSC suspensions.

## Introduction

Mesenchymal stem cell (MSC) therapies have been frequently investigated as effective stem cell therapies to address diverse pathologies since MSCs secrete diverse cytokines involved in cell proliferation, neoangiogenesis, inflammatory suppression, and immunoregulation [1–5]. MSCs from various tissues sources, including bone marrow, amniotic fluid, adipose tissue, dental pulp, and umbilical blood are applied in cell therapies [6]. Among these, human umbilical cord-derived mesenchymal stem cells (hUC-MSCs) have attracted recent attention for their higher proliferative capacity and cell viability compared to bone marrow or adipose tissue-derived MSCs [7–11]. Moreover, UC-MSCs secrete higher levels of therapeutic cytokines compared to the other two MSC types [8]. In addition, hUC-MSCs are obtained noninvasively from umbilical cords obtained postnatally as routine tissue discards from childbirth [12].

To exploit numerous MSC therapeutic benefits in vivo, cell administration and transplantation using cell suspension injections, both systemically and locally, and direct tissue transplantation of MSCs within various scaffolds and carriers have been extensively investigated. Nonetheless, administration of MSCs to humans clinically exhibits the requisite safety but generally fails to yield convincing therapeutic benefits observed in preclinical in vitro and in vivo models [7].

Despite over 900 MSC clinical trials describing these MSC delivery strategies for a multitude of different therapeutic goals, results are inconsistent [13], with only 3 human MSC therapies approved for immune-related diseases (i.e., graft-versus-host disease) globally to date (none approved in USA) [14]. Given these long-standing challenges to effective MSC therapies, current standard MSC dosing and administration practices utilizing suboptimal injection-based delivery of “naïve” (i.e., heterogeneous, non-standardized) MSC suspensions or carrier-based constructs, both autologous and allogenic, should be reconsidered and redesigned around critical quality attributes and delivery methods [15–21].

MSC sheets—contiguous, robust, viable monolayers of cultured MSCs—have been reported as an alternative implantable cell therapy delivery system [22–25]. Cell sheets are fabricated using thermoresponsive poly(*N*-isopropylacrylamide) (PNIPAAm)-modified commercial cell culture surfaces. PNIPAAm exhibits reversible hydration/dehydration changes in response to cell culture temperatures near 37°C [26–37], facilitating cell harvesting from culture as versatile single, scaffold-free sheets useful for cell therapy and tissue engineering [38–45]. Cell sheets harvested without use of destructive enzymes retain functional cell–cell junctions and endogenous extracellular matrix (ECM), unlike cells recovered using trypsin digestion [40]. Furthermore, sheets are readily handled and transplanted into patients, either ectopically or orthotopically without suturing, exploiting innate tissue adhesion enabled by sheet ECM.

MSC sheet transplantation is interesting for regenerative medicine applications due to known enhanced production of therapeutic cytokines over MSC suspensions [23, 46–48]. Most MSC sheet applications have used bone marrow- or adipose tissue- derived MSC sheets; UC-MSCs are more recently reported in MSC sheets. hUC-MSC sheets have been characterized in vitro by observing cellular microstructures, ECM, cell–cell junctions, cell–ECM junctions, layered sheet constructs, and various cytokine and chemokine production [49–53]. Further, hUC-MSC sheet interactions with tissue culture surfaces have been investigated in vitro [8]. Despite increasing hUC-MSC sheet in vitro analysis, hUC-MSC sheet tissue interactions after transplantation in vivo, including sheet engraftment behavior and in situ cytokine secretion critical to their therapeutic relevance, has not yet been reported.

The aim of this study was to compare hUC-MSC sheets and two types of analogous cultured hUC-MSC suspensions in vivo in a murine subcutaneous tissue site to elucidate their respective in vivo therapeutic signals via local cell engraftment and cytokine production.

## Materials and methods

### Human umbilical cord MSC culture

Human MSCs derived from umbilical cord Wharton’s jelly were obtained from PromoCell (Heidelberg, Germany). hUC-MSCs were cultured in Dulbecco’s modified Eagle’s medium (DMEM) (Gibco, Waltham, MA, USA) supplemented with 10% fetal bovine serum, 1% MEM nonessential amino acids, 1% GlutaMAX, 100 units/mL penicillin, and 100 μg/mL streptomycin. hUC-MSCs were incubated at 37°C with 5% CO_2_ in a humidified chamber and passaged after cells reached confluence on 10-cm-diameter culture dishes (Thermo Fisher Scientific, Waltham, MA, USA). In passage culture, hUC-MSCs were recovered through a digestive proteolytic enzyme (TrypLE, Gibco) treatment for 5 min and seeded into other standard tissue culture dishes (Thermo Fisher Scientific) at a density of 4000–6000 cells/cm^2^; hUC-MSCs at passage 3–6 were used in this study.

### Preparation of hUC-MSC sheets and suspensions

**Fig. 1:**
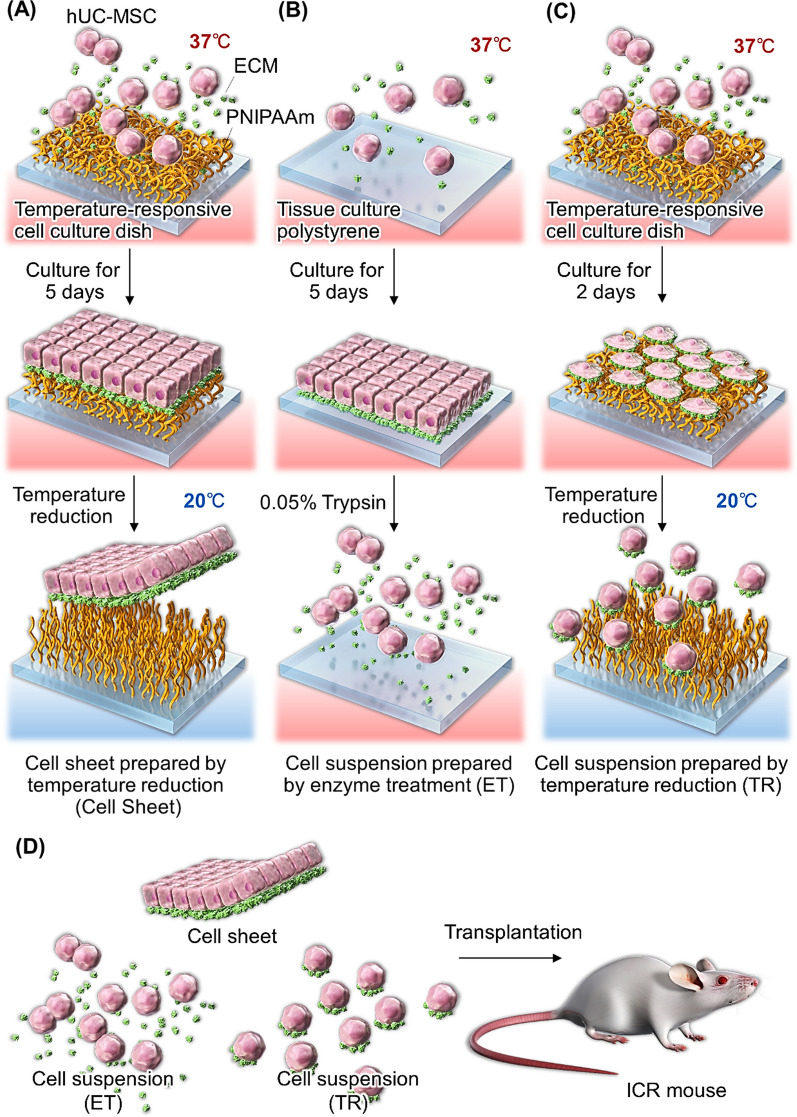
Schematic illustration for preparing (**A**) a human umbilical cord-derived mesenchymal stem cell sheet using a temperature-responsive cell culture dish; (**B**) a conventional cell suspension after enzyme treatment; and (**C**) a cell suspension by temperature treatment in a temperature-responsive cell culture dish; (**D**) transplantation of cell sheets or two different cell suspensions to the mouse.

hUC-MSC sheets were prepared by the following procedure (Fig. 1): Cultured hUC-MSC were recovered from conventional 10-cm-diameter culture dishes using TrypLE treatment for 5 min. Then, recovered hUC-MSCs were seeded into 35-mm-diameter temperature-responsive culture dishes (UpCell™, CellSeed, Tokyo, Japan) at a density of 2 × 10^5^ cells/dish. Next, hUC-MSCs were incubated until confluency for 5 days in DMEM before moving these culture dishes to an incubator set at 20°C, and incubating for 30 min. At this point, the hUC-MSC sheets spontaneously release from the culture surfaces and are recovered from these dishes as single units [54]. Cell sheets contained approximately 4 × 10^5^ cells [8].

Two types of hUC-MSC suspensions were prepared as follows: hUC-MSCs were seeded on conventional 35-mm-diameter cell culture dishes (Thermo Fisher Scientific) and incubated using DMEM for 5 days until confluency. Then, hUC-MSC suspensions were recovered from culture using 0.05% trypsin for 5 min. This harvested cell suspension was called as “enzyme treatment (ET).” Another cell suspension was seeded in 10-cm-diameter temperature-responsive cell culture dishes (UpCell™) in DMEM and incubated for 48 h (i.e., below cell confluence). Then, hUC-MSC suspensions were recovered from these dishes by changing culture temperature from 37°C to 20°C. Individual cells were released from these surfaces after 30 minutes and collected. This recovered cell suspension was called “temperature reduction (TR).” Cell counts in each suspension were approximately 4 × 10^5^ cells.

### Subcutaneous transplantation of hUC-MSC sheets and cell suspensions

The reporting of all animal experiments adheres to the ARRIVE guidelines. The Institutional Animal Care and Use Committee at Keio University approved this procedure. Female Crl:CD1 (ICR) mice (6–8 weeks old, Japan SLC, Hamamatsu, Japan) were used in this study. A murine disease model is frequently used to monitor and validate therapeutic efficacy of the transplanted cells; however, in this study we used a healthy mouse transplant model. This is because this study sought to compare in vivo survival and in vivo cytokine production of hUC-MSC sheets and two types of hUC-MSC suspensions, and not to assess any therapy or disease efficacy. Therefore, murine cell transplant experiments used healthy instead of diseased murine models in order to eliminate factors that might otherwise possibly confound interpretation of cell transplantation effects only. Mice were housed in a specific pathogen-free facility and fed alfalfa-free food (AIN-76A Rodent Diet, D10001; Research Diets, New Brunswick, USA) during the week before transplantation to reduce tissue autofluorescence sourced from the feed. The locations of all mouse cages were randomized to avoid confounding bias.

**Fig. 2:**
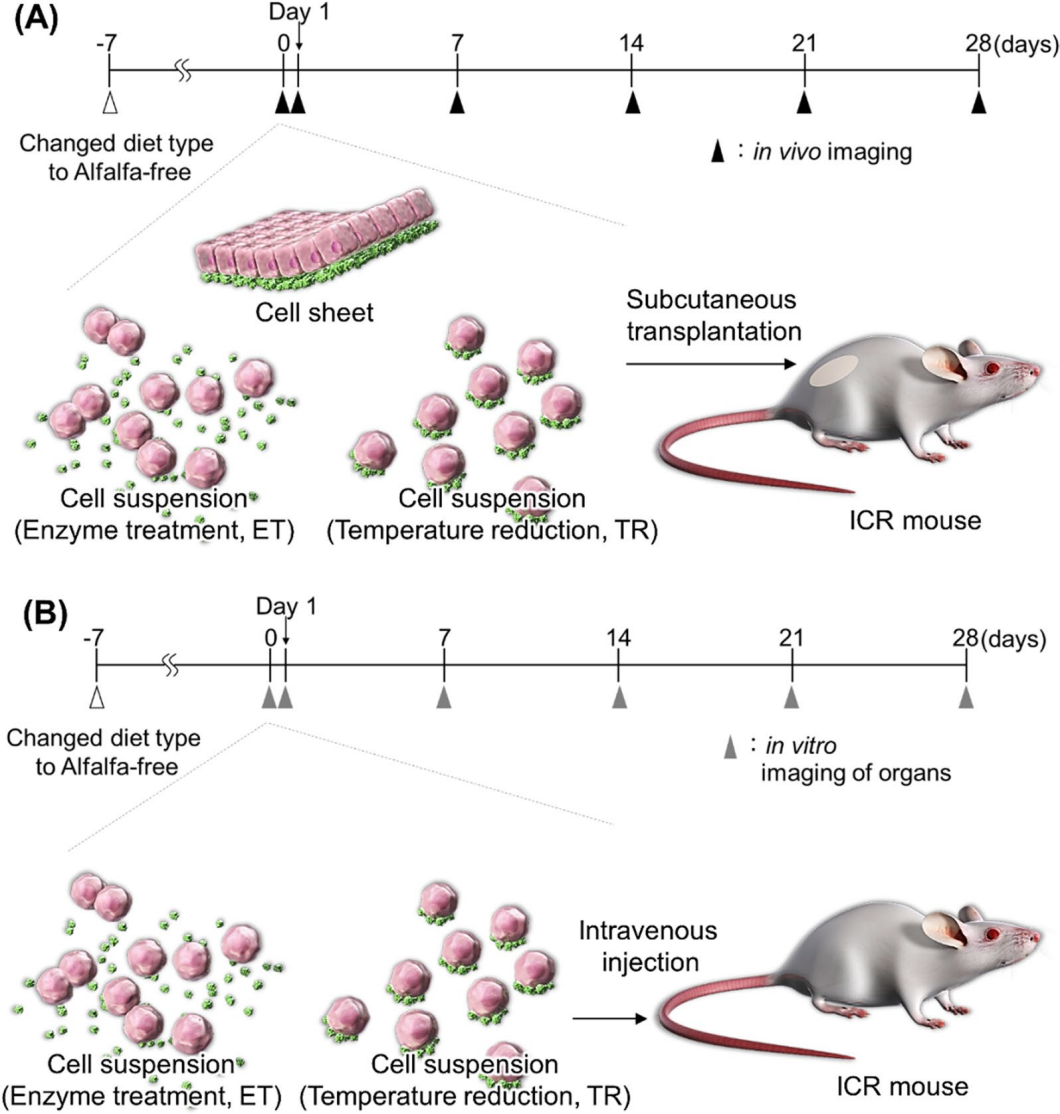
Illustration of the cell transplantation and imaging techniques used for assessing the human umbilical cord-derived mesenchymal stem cell sheets and suspensions. (**A**) Subcutaneous transplantation of sheets and suspensions; (**B**) intravenous tail vein injection of cell suspensions.

In this investigation, the following two forms of hUC-MSC transplantation were performed: intravenous cell suspension injection and subcutaneous sheet transplantation (Fig. 2). Three different transplantation groups—hUC-MSC sheets, ET hUC-MSC suspensions, and TR hUC-MSC suspensions—were transplanted subcutaneously (Fig. 2A). An additional sham control group was used. Two transplantation groups—ET hUC-MSC suspensions and TR hUC-MSC suspensions—were performed through intravenous injection (Fig. 2B). To assess the effectiveness of cell engraftment and cytokine expression, eight mice were employed per group, established by accounting for the reduction of errors related to individual variations in mice. For the duration of the cell transplant trial, a total of 48 mice were used. To prevent confounding bias, the placement of each mouse cage was randomized following cell transplantation.

hUC-MSC sheets were stained with fluorescent dye XenoLight DiR (DiIC18(7))(1,1′-dioctadecyltetramethylindotricarbocyanine iodide, Caliper Lifesciences, Hopkinton, USA) after 1-h incubation. Both hUC-MSC suspensions were stained with this same dye by a 30-min incubation. The difference in cell staining duration is due to different stain uptake efficiencies between cell sheet and cell suspension. hUC-MSC suspensions were stained with a fluorescent dye for 30 min, sufficient for saturated staining of the cell suspension in vivo. By contrast, cells within cell sheets do not stain consistently with fluorescent dye when compared with cell suspensions. Therefore, a longer staining duration (60 min) is required for saturated staining of cell sheets. This change in staining does not influence resulting fluorescent intensities because each fluorescent intensity is maximally saturated with each staining duration.

hUC-MSC sheet transplantation was performed using the following procedure. An ICR mouse was anesthetized with somnopentyl and the hair on its back removed. Then, an incision in the dorsal skin was lifted to expose the subcutaneous tissue. One side of a silicone membrane was sutured to the interior surface of the exposed skin. The fluorescently labeled hUC-MSC sheet was placed between the skin and the fixed silicone membrane, and then replaced gently with the membrane directly against the subcutaneous tissue surface, and the incision was closed with 5-0 nylon sutures. After 1 h of transplantation, and at 1, 7, 14, and 28 days, fluorescent images of the mouse were acquired noninvasively using an in vivo imaging system (IVIS Spectrum, Caliper Lifesciences) at 710-nm excitation, 760-nm emission. During image acquisition, the mouse was anesthetized with isoflurane. Images were analyzed using IVIS Living Image (Caliper Lifesciences).

Two types of fluorescently dyed hUC-MSC suspensions, “ET” or “TR” (see Figures 1 and 2), were transplanted into ICR mice in identical subcutaneous locations by subcutaneous injection as follows: ICR mice were anesthetized with isoflurane, and a given cell suspension containing 4 × 10^5^ cells in DMEM was injected into dorsal subcutaneous tissues (Fig. 2A). After 1h of transplantation and at 1, 7, 14, and 28 days, fluorescent images were acquired noninvasively using IVIS in vivo imaging system, identically to the process for imaging cell sheets in vivo.

Two types of fluorescently dyed hUC-MSC suspensions, “ET” or “TR,” was transplanted into ICR mice by intravenous injection as follows: ICR mice were anesthetized with isoflurane, and a cell suspension containing 4 × 10^5^ cells was injected into the tail vein. After transplantation for 60 min, 1, 7, and 14 days, mice were anesthetized with isoflurane and euthanized by cervical dislocation and their organs, brain, heart, kidney, stomach, lungs, spleen, and liver, were collected. Fluorescent images of the collected organs were obtained using IVIS imaging.

### Cytokine assay from hUC-MSC sheets and cell suspensions in vivo

Human cytokines secreted by UC-MSCs in the subcutaneous transplant site or in murine plasma were measured by enzyme-linked immunosorbent assay (ELISA). Expression levels of the VEGF (vascular endothelial growth factor), HGF (hepatocyte growth factor), TGF-β1 (transforming growth factor beta 1), IL-10 (interleukin 10), and IL-6 (interleukin 6) were measured by ELISA. At 28 days post-transplantation, each mouse was anesthetized with isoflurane and its blood was collected from cardiac puncture. The mouse was then euthanized via cervical dislocation. The collected blood was centrifuged at 3000 rpm for 10 min, and the supernatant plasma was collected and frozen at − 80 °C until cytokine concentration assay was performed. After blood collection, dorsal subcutaneous tissues were also collected and placed in RIPA buffer (Wako, Osaka, Japan), with a protease inhibitor cocktail (Nacalai Tesque, Kyoto, Japan) added, and minced using scissors. Then, the tissue was sonicated at 4 °C for 10 min and repeated freeze–thawing of the tissue suspension (x3) was conducted to extract proteins, and stored at − 80 °C until cytokine concentration assay. Cytokine concentrations of the prepared plasma and tissue extract media suspension were quantified using a human cytokine ELISA assay kit (R&D Systems, Minneapolis, MN, USA). Protein concentration of each sample for ELISA was normalized by measuring plasma protein concentration with a BCA Protein Assay Kit (Thermo Fisher Scientific). Samples collected from mice without any hUC-MSC transplantation were used as negative control.

### Statistical analysis

All values are expressed as average values and standard error (mean ± SEM). Differences were analyzed by Tukey’s test for multiple samples and by the Student’s t test for two groups. *p < 0.05 or **p < 0.01 were considered significant.

## Results and Discussion

### hUC-MSC engraftment duration in murine subcutaneous transplantation

**Fig. 3:**
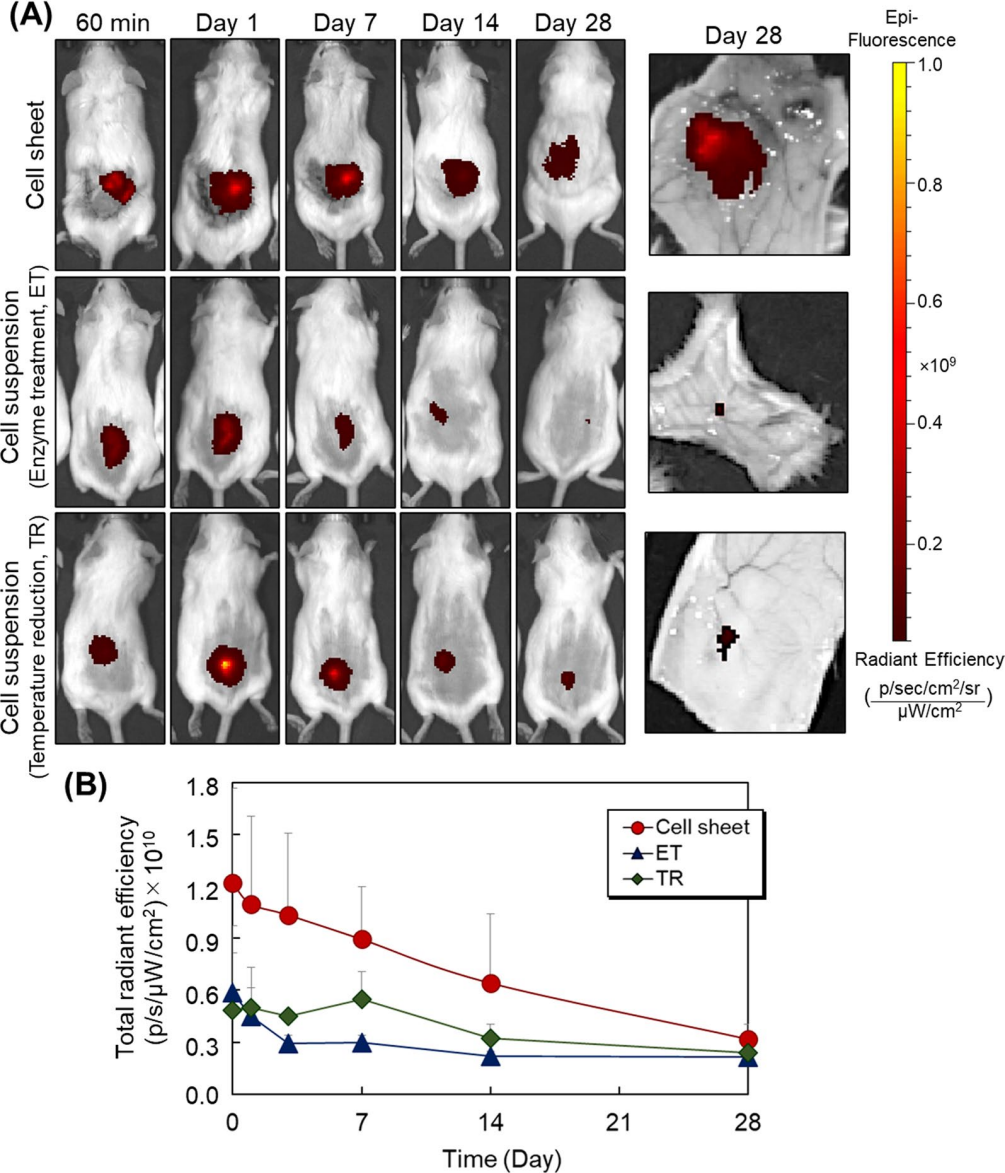
Optical evaluation of the engraftment of transplanted mesenchymal stem cell sheets and suspensions prepared from human umbilical cord sources in an ICR mouse. Subcutaneous transplants of human cell sheets and cell suspensions (ET or TR) were performed (see Fig. 2). (**A**) In vivo imaging of cell survival during cell sheet transplantation and cell suspension injection (ET and TR). (**B**) A quantitative evaluation of the in vivo fluorescence intensity of transplanted cell sheets and cell suspension (ET or TR)

hUC-MSC sheets and two types of hUC-MSC suspensions were prepared in cultures and harvested using either a commercial temperature-responsive cell culture surface or traditional enzymatic treatment with trypsin (Fig. 1). All MSCs were stained with fluorescence dye and transplanted into the dorsal subcutaneous tissues of ICR mice (Fig. 2A). Engraftment of transplanted hUC-MSC was monitored using an in vivo fluorescent imaging system at 60 min, and at 1, 7, 14, and 28 days (Fig. 3). At 28 days post-transplantation, the dorsal subcutaneous tissues were collected and hUC-MSC engraftment was observed by IVIS imaging (Fig. 3A). At 60 min and 1 day after transplantation, hUC-MSC engraftment was similar across all three MSC transplantation models. These results indicate that transplanted hUC-MSCs can survive in immune-competent murine host subcutaneous tissue at least until 1 day post-transplantation regardless of the cell preparation/implantation method.

hUC-MSC sheets were retained continuously in subcutaneous engraftment up to 28 days post-transplantation (Fig. 3A). In contrast, ET MSC suspension injections exhibited decreased engraftment at 7 days, nearly disappearing at 28 days. Similarly, the TR MSC suspension injection also exhibited reduced engraftment over time. Distinct hUC-MSC engraftment duration was attributed to the endogenous MSC ECM and cell–cell junctions known to be retained for hUC-MSC sheet preparations [22, 53]. Improved MSC immunomodulatory properties in sheets versus dispersed cells might also contribute to this improved xenogenic MSC sheet retention [22]. Previously, endothelial and kidney cell sheets were also shown to maintain ECM and adhesive functions after culture recovery, with ECM fibronectin displayed basally under cell sheets [40, 55]. Furthermore, hUC-MSC sheets retain both structure and integrated function, including their ECM, cell–cell junctions, and cell–ECM junctions after culture harvest [49, 53]. This explains the strong spontaneous adhesion and retention in tissue sites without suturing observed for transplanted hUC-MSC sheets. In contrast, ET hUC-MSC suspensions lost both their ECM and cellular activity and could not effectively engraft into host subcutaneous tissue. However, the TR hUC-MSC suspension exhibited higher cell engraftment in vivo than the ET suspension as it retains its endogenous ECM and other surface proteins without proteolytic treatment.

These results support higher in vivo retention duration for xenogenic hUC-MSC sheet implants than both hUC-MSC suspension injections due to sheet integrity, ECM and cell–cell junctions retained from culture and harvest processing, and possibly enhanced MSC sheet immunomodulatory potential with host subcutaneous tissue compared to isolated injected MSCs.

As previous reports have indicated that tumorigenesis from transplanted human mesenchymal stem cells is negligible in an in vivo model [56–60], this risk at 28 days post-transplant was considered to be negligible.

### Engraftment duration of hUC-MSCs after intravenous injection

**Fig. 4:**
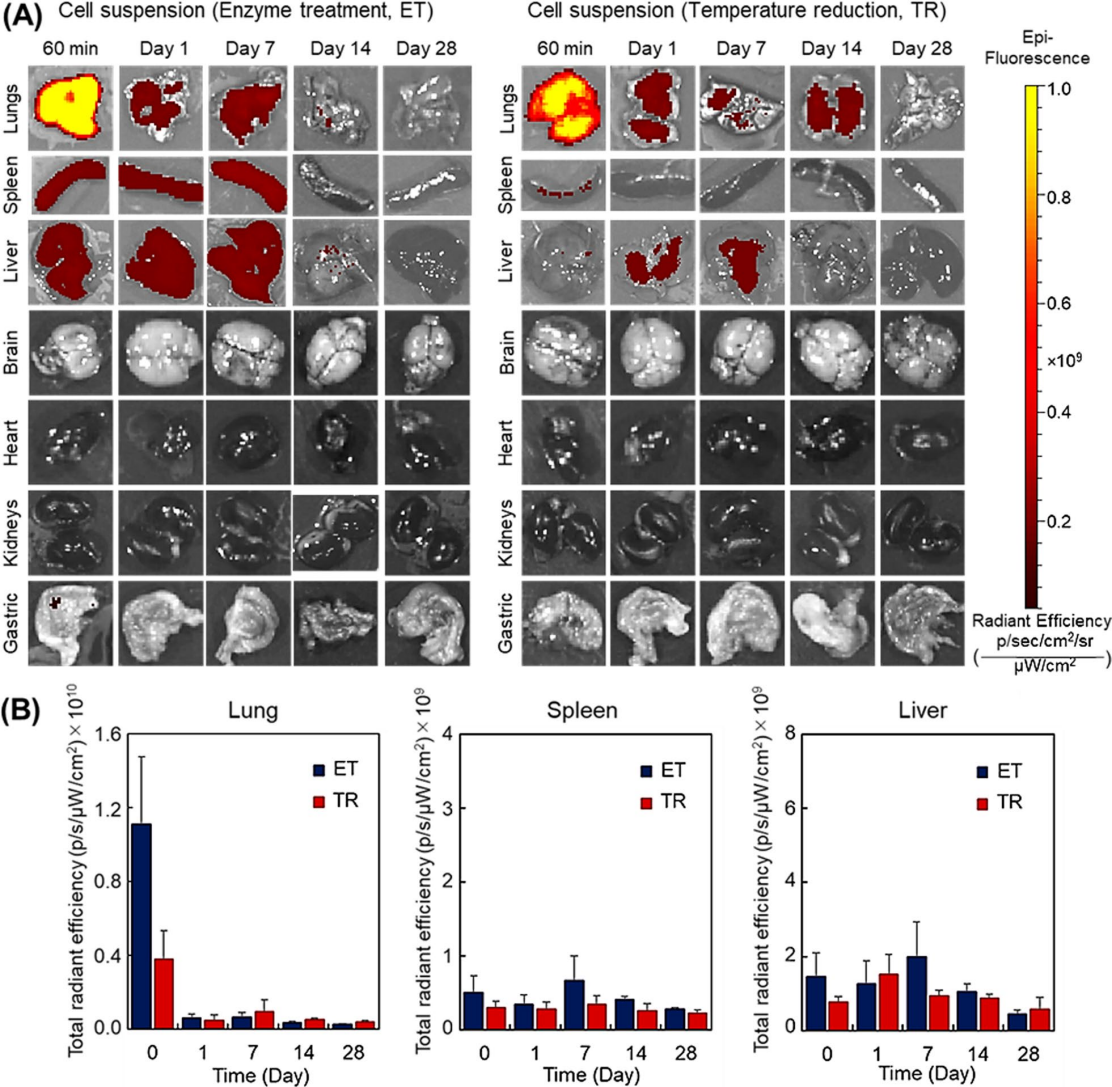
Analysis of the engraftment of human umbilical cord-derived mesenchymal stem cell suspensions prepared by either enzyme treatment (ET) or temperature reduction (TR). (**A**) IVIS imaging of cell engraftment in various organs. (**B**) Quantitative analysis of IVIS fluorescent intensity in various organs.

In most clinical cases, MSCs cell suspensions have been introduced via intravenous or direct tissue injection. Thus, the two types of hUC-MSC suspensions were intravenously injected, and MSC engraftment efficiency in various organs was investigated (Fig. 4). For the ET hUC-MSC suspension, at 1 h after intravenous infusion, substantial cell engraftment was observed in the lung, and an even larger fraction in the spleen and liver. At 1 day post-infusion, the number of hUC-MSCs remaining in the lung notably decreased. Up to 7 days post-infusion, hUC-MSCs were present in the lung, spleen, and liver. However, at 14 days post-infusion, most fluorescent hUC-MSCs had cleared, indicating that hUC-MSC ET suspensions would only be present and active primarily in the lung, spleen, and liver up to 7 days post-infusion.

TR hUC-MSC suspension intravenous infusions produced similarly substantial hUC-MSC retention in the lung at 1 h post-infusion, while hUC-MSC presence in other organs was not observed (Fig.4). At 14 days post-infusion, hUC-MSCs were still observed in the lung, indicating that the TR hUC-MSC suspension infusion is retained longer in the lung (14 days) than the ET hUC-MSC suspension (7 days).

Compared with subcutaneous hUC-MSC tissue cell suspension injections, intravenous MSC infusions exhibited relatively low retention duration (compare Figures 3 and 4). Thus, intravenous MSC injection appears to be a less effective hUC-MSC administration or dosing method, similar to low infused MSC tissue site engraftment and retention observed in clinical studies [19].

### Cytokine expression level comparisons of transplanted hUC-MSCs

**Fig. 5:**
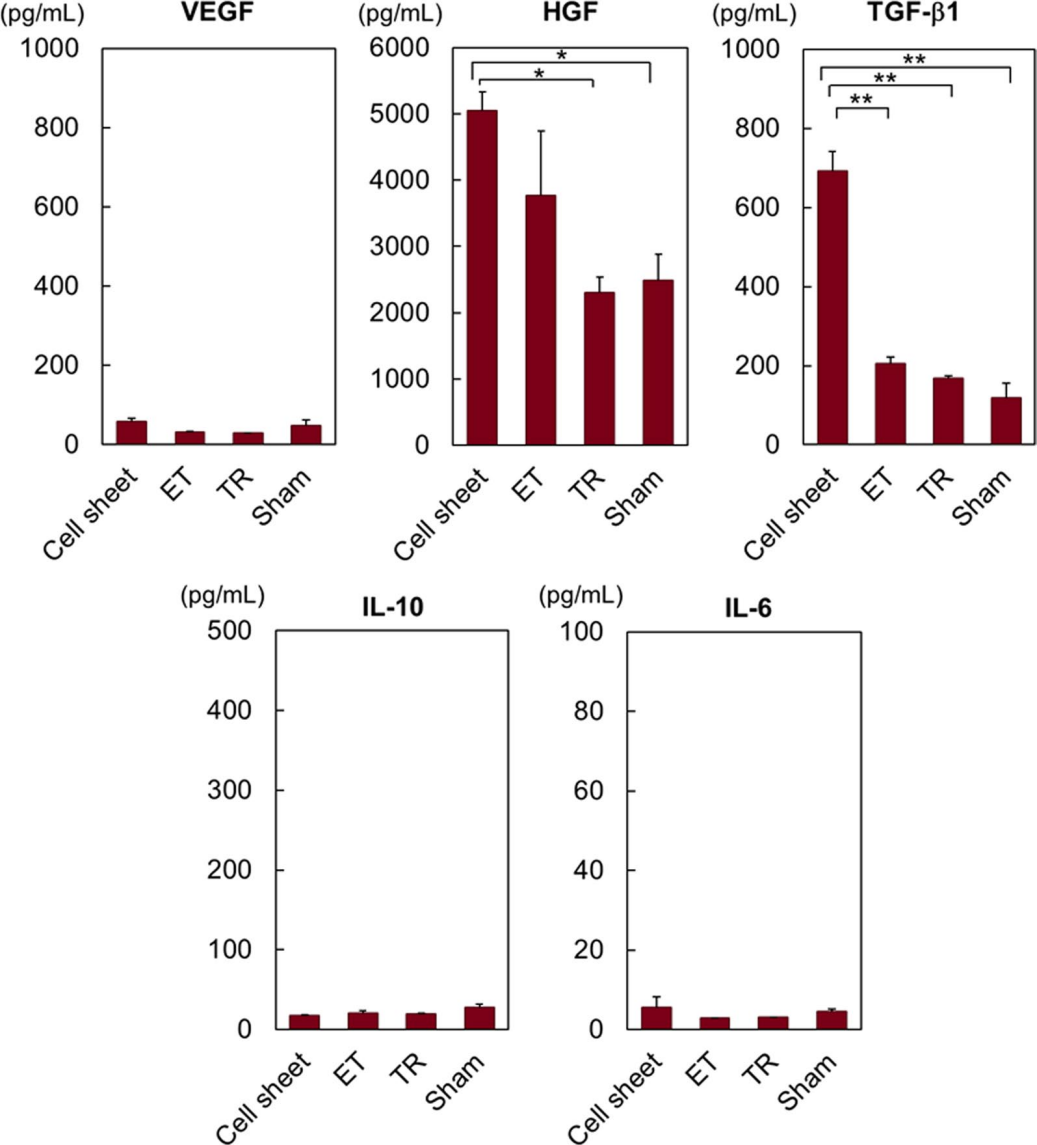
Human cytokine expression profiles in human MSC transplant sites in mice. Comparisons of subcutaneously transplanted human mesenchymal stem cell sheets and cell suspensions cord (ET or TR), all derived from human umbilical sources. Secreted cytokine levels in the subcutaneous human umbilical cord-derived mesenchymal stem cell sheet transplant site are examined using ELISA. Levels of VEGF, HGF, TGF-β1, IL-10, and IL-6 were measured. The subcutaneous region of sham-treated ICR mice was used as a negative control. (*n* = 3) * *p* < 0.05; ** *p* < 0.01.

**Fig. 6:**
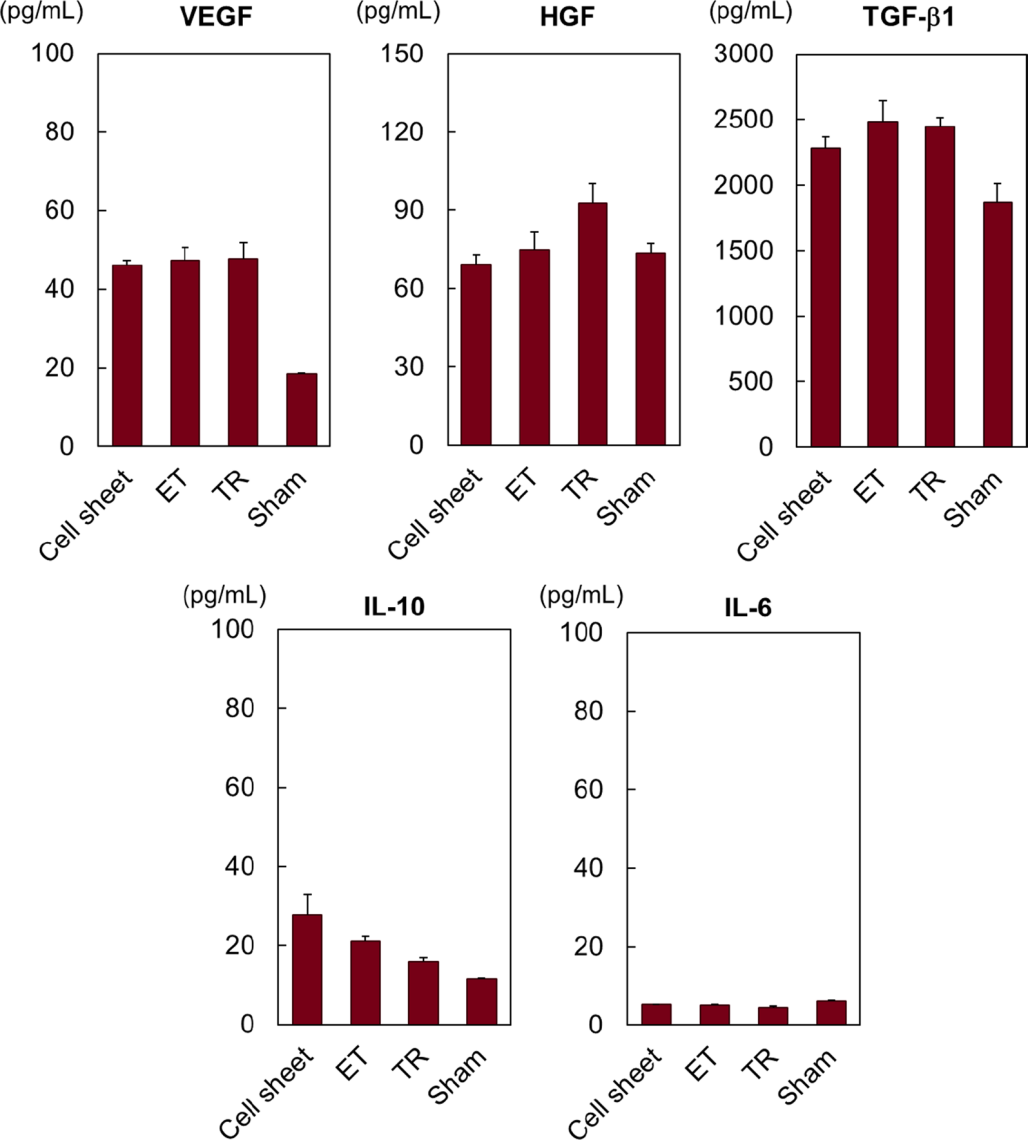
Human cytokine expression in murine blood samples after subcutaneous transplants of human umbilical cord-derived mesenchymal stem cell sheets and injected cell suspensions (ET or TR). Secreted human cytokine levels of VEGF, HGF, TGF-β1, IL-10, and IL-6 in blood were evaluated by ELISA. Sham-treated ICR mice were used as a negative control (*n* = 3)

Previous reports consistently indicate that hUC-MSCs secrete various therapeutic cytokines *in vitro* including HGF and TGF-β1 [8, 23, 24, 50–53]. However, cytokine secretion after in vivo hUC-MSC transplantation has not been reported. Hence, we measured human cytokine levels at both the MSC transplant tissue site and in blood at 28 days post-transplantation (Figs. 5 and 6). At the subcutaneous transplant site, hUC-MSC sheets exhibited higher HGF and TGF-β1 levels compared to those of both transplanted ET and TR cell suspensions (Fig. 5). This can be explained by higher MSC retention and engraftment duration for hUC-MSC sheets at these tissue sites (Fig. 3). HGF enhances cell viability, proliferation ability, migration capability, and inflammatory suppression [61–64] and has shown positive effects as treatment for multiple sclerosis and COPD [65, 66]. Thus, possible HGF therapeutic effects might be enhanced by transplantation of hUC-MSC sheets versus MSC suspension injections. In addition, TGF-β1 produces inflammatory suppression by inhibiting macrophage activation and secretion of inflammatory cytokines [61, 67, 68]. Thus, hUC-MSC sheets might better reduce host inflammatory response at cell transplant sites, even better assisting host xenogenic immunomodulation and human MSC tolerance in this murine model, as manifested by enhanced MSC sheet retention and reported sheet immunomodulatory phenotype [22]. In contrast, no obvious difference in human VEGF, IL-10, and IL-6 levels are detected, indicating that neither hUC-MSC sheets nor cell suspensions might be effective in these specific cytokine-mediated in vivo effects.

Human cytokine levels in blood were measured at 28 days after subcutaneous MSC transplantation (Fig. 6). No differences in levels of any tested cytokine between groups are observed, probably because they are either absent, or sufficiently dilute in blood to remain below the ELISA assay detection limit in blood. This indicates that hUC-MSC transplantation affects only the local tissue transplant environment, using paracrine signaling rather than endocrine effects. This result has important implications for possible future applications in MSC cell sheet therapy.

These collective results demonstrate that surgically placed hUC-MSC sheets remain engrafted longer and in higher viable cell numbers in tissue implant sites than injected hUC-MSC suspensions. In addition, hUC-MSC sheets in vivo secrete higher levels of select cytokines to local tissue sites than either injected hUC-MSC suspension. Thus, hUC-MSC sheet transplantation represents an improved alternative approach for enhancing MSC-based cell therapies based on this preclinical murine xeno-transplant in vivo analysis.

## Conclusions

We report comparisons for murine subcutaneous engraftment of surgically placed subcutaneous hUC-MSC sheets and injected cell suspensions after transplantation, and resulting select human cytokine secretion profiles in vivo resulting from transplanted hUC-MSC. Subcutaneous hUC-MSC sheets exhibit higher engraftment with longer retention compared to hUC-MSC suspensions prepared either by conventional enzyme treatment or harvested by temperature reduction without trypsin treatment. This difference is attributed to retention of endogenous ECM and cell–cell junctions in sheets but not in suspension forms, and perhaps also improved immunomodulatory properties of human MSC sheets versus their suspensions in this murine xenograft model. Human cytokine secretion from each form of transplanted hUC-MSC to local subcutaneous tissue and to systemic blood circulation was also investigated. Significant amounts of HGF and TGF-β1 were observed in local tissue sites transplanted with hUC-MSC sheets compared to sites bearing either hUC-MSC suspension. This difference may be attributed to longer retention of larger amounts of MSCs in sheet-transplanted sites at assay time points. On the contrary, no intergroup differences were observed in levels of any tested cytokines in blood. This indicates that hUC-MSC transplantation only affects the local tissue environment at transplant sites, using paracrine signals rather than endocrine effects. This result has important implications for possible future applications in MSC cell sheet therapy. If these xenogenic murine cell implant model data reflect human allogenic MSC results, then transplantation of hUC-MSC sheets may offer a more effective therapeutic approach due to enhanced, local MSC engraftment with more substantial, more sustained secretion of human cytokines useful for various cell therapies and regenerative medicine.

## Data Availability

The datasets used and/or analyzed during the current study are available from the corresponding author on reasonable request.

## Abbreviations

COPD: Chronic obstructive pulmonary disease
DMEM: Dulbecco’s modified Eagle’s medium
ECM: Extracellular matrix
ELISA: Enzyme-linked immunosorbent assay
ET: Enzyme treatment
HGF: Hepatocyte growth factor
hUC-MSC: Human umbilical cord-derived mesenchymal stem cell
IL-6: Interleukin 6
IL-10: Interleukin 10
IVIS: In vivo imaging system
MEM: Minimum essential medium
MSC: Mesenchymal stem cells
PNIPAAm: poly(*N*-isopropylacrylamide)
TGF-β1: Transforming growth factor beta 1
TR: Temperature reduction
VEGF: Vascular endothelial growth factor
